# Super and deepened-extinction in human predictive learning and a comparison of associative models

**DOI:** 10.3758/s13420-025-00681-4

**Published:** 2025-07-15

**Authors:** Ovidiu Brudan, Hedwig Eisenbarth, Steven Glautier

**Affiliations:** 1 Southampton University, Southampton, England; 2 Victoria University of Wellington, Wellington, New Zealand

**Keywords:** Associative learning, Extinction, Rescorla–Wagner, Pearce, Configural, Relapse, Response recovery, Maximum likelihood, Akaike information

## Abstract

Cue-exposure is a treatment (e.g. for addictions and phobias) that aims to extinguish conditioned responses to target cues. However, especially in the case of addiction, relapse still occurs after cue-exposure and this may be due to recovery of conditioned responses outside of the extinction context. Super-extinction and deepened-extinction are two compound-cue extinction procedures which have been assessed for their capacity to produce more robust extinction than standard single-cue extinction procedures. We carried out further assessment of super and deepened-extinction protocols but found no evidence that they produced less response recovery compared to single-cue extinction. Contrariwise, super-extinction actually produced more recovery than the other two conditions. These results can be understood in terms of configural associative models (configural Rescorla–Wagner and Pearce configural model) but not in terms of the simple elemental Rescorla–Wagner model. Furthermore, the configural models provided better fits to overall data, and the Pearce configural model was better than the configural Rescorla–Wagner model.

Associative learning allows organisms to adapt to their environments and is a basic survival mechanism conferring an evolutionary advantage. Perhaps one of the most important features of associative learning is its flexibility. Once learning has taken place, it can be changed through various routes if environmental changes lead to that learning becoming outdated or even maladaptive. One such route is extinction. A simple extinction procedure involves presenting a previously trained conditioned stimulus (CS) without the unconditioned stimulus (US) it was previously associated with. As a result of these ‘non-reinforced’ presentations of the CS the conditioned response (CR) generated by the CS diminishes. However, as discussed below, phenomena such as recovery and renewal show that extinction involves more than simply unlearning a previously learnt association (e.g. Bouton, 1993; 1994; 2000) and this has provided challenges to the various models of associative learning that have been developed. Furthermore, re-emergence of extinguished CRs has been identified as a potential limiting factor in applied settings where the goal of cue-exposure treatment is to extinguish CRs to e.g. drug-related cues. In what follows, we introduce response recovery and renewal of extinguished responding alongside theoretical and applied considerations before outlining the two primary objectives of the current investigation namely 1) to compare standard, super, and deepened extinction procedures and 2) to compare associative models of these procedures.

Recovery refers to the re-emergence of an extinguished CR when the CS is re-presented following a delay after extinction. On the other hand, renewal refers to the re-emergence of an extinguished CR when the CS is presented in a context that differs from the context in which extinction took place. However, these traditionally identified distinctions may be more apparent than real because both of these phenomena can potentially be explained through mechanisms involving contextual stimuli, with recovery being treated as a special case of renewal in which the passage of time implicitly modifies the context. In the case of renewal contextual changes are explicit e.g. when the environment changes after extinction. Therefore, in what follows, we use the terms recovery and renewal interchangeably. Contextual stimuli are those that remain constant across the course of multiple learning trials and can be contrasted with the punctate CSs and USs that mark the learning trials. One approach to explaining renewal is through conditioned inhibition and ‘protection-from-extinction’. According to this, based upon the Rescorla–Wagner associative model (Rescorla & Wagner, 1972), when the context changes during extinction, the new context behaves as a CS and acquires inhibitory properties. Therefore, post-extinction, when the CS is presented outside of this context, a renewal effect may occur because the inhibitory influence of the extinction context is no longer present. Some experiments have shown that extinction carried out in the presence of a discrete inhibitory stimulus can protect from extinction (e.g. Rescorla, 2003), but the evidence for contextual stimuli functioning in that way is mixed (e.g. Bouton & Swartzentruber, 1986; Glautier et al., 2013; Polack et al., 2012).

Another associative model, developed by Pearce (1994), can also explain renewal but the mechanism differs from that proposed in the Rescorla–Wagner model. The Rescorla–Wagner model is an elemental model, treating the CSs involved in conditioning as discrete elements each of which can enter into associations with USs. In contrast, Pearce’s model is a configural model in which the discrete stimulus elements encountered on each learning trial form ‘configurations’ and the configurations themselves are the candidates for forming associations with USs. Renewal in the Pearce configural model is determined by the similarity relations between the stimulus configuration used in the post-extinction test and the other stimulus configurations previously encountered during acquisition and extinction. Renewal occurs if the net of generalised excitatory and inhibitory influences produced by the post-extinction test configuration is greater than zero.

It is of practical and theoretical interest to get a better understanding of the mechanisms underlying extinction. On the practical side, there are therapeutic interventions based on extinction which could be improved. In the case of addiction, it has long been accepted that relapse is a major problem with typically less than 50% ‘survivors’ three months after initiating abstinence and this applies across a range of substances and even in individuals receiving clinical interventions (e.g. Anton et al., 2006; Fortmann & Killen, 1995; Northrup et al., 2015). Cue-exposure for addiction is based on an underlying model of addiction in which drug-related stimuli – drug-cues, become CSs because they are repeatedly paired with drug USs. The CRs produced by drug-cues are thought to play a part in relapse, and cue-exposure treatment aims to reduce relapse risk by extinguishing CRs to drug-cues by repeated presentation of the cues without a drug US. Unfortunately, although cue-exposure is effective for treatment of some conditions (e.g. phobias cf. Choy et al., 2007), its effectiveness in the treatment of addiction is not well established, but a small number of studies suggest it is an intervention worthy of further investigation (e.g. Kiyak et al., 2023).

Renewal effects may be one factor that limits the effectiveness of cue-exposure treatments (e.g. Bouton, 2000; Conklin & Tiffany, 2002) and some experiments have provided evidence that carrying out extinction in multiple contexts may reduce renewal effects (e.g. Bustamante et al., 2016; Glautier et al., 2013). An alternative approach, which is the focus of the current paper, is to carry out extinction in the presence of multiple excitatory cues (e.g. Craske et al., 2014). The objective of carrying out extinction in the presence of multiple excitatory cues is to increase the amount of associative change that occurs during extinction. According to associative models, such as the Rescorla–Wagner and the Pearce configural models introduced above, associative change is driven by prediction error. An error signal is generated during extinction because a cue that has previously signalled an outcome is presented in the absence of that outcome. It follows from these associative models that if the prediction error can be increased during extinction, then the amount of learning during extinction will be correspondingly increased. One way to increase prediction error, instead of presenting single cues on each extinction trial, is to present compounds of multiple excitatory cues on each trial during extinction. To explain this further, the Rescorla–Wagner and the Pearce configural models both make use of an error term given in Eq. 1.

1 $$\begin{aligned} \lambda -\sum V \end{aligned}$$

In Eq. 1 the value of $\lambda$ is used to indicate the status of the US on each learning trial. We set $\lambda =1$ when there is a US, as in acquisition, and we set $\lambda =0$ when there is no US, as in extinction. The subtrahend, $\sum V$, represents the summed associative strength of all cues present on that trial. So, in a simple case for the Rescorla–Wagner model, assuming cue A has been trained to asymptote during an acquisition phase then we would have $V_A\rightarrow \ 1$ and then we begin extinction of cue A. On the first extinction trial $\sum V=V_A$ since cue A is the only cue present and the error on this first extinction trial would therefore approach -1 and this value determines the amount of associative change for cue A. Now if, during acquisition, cues A and B had both been trained to asymptote we would have the option presenting an AB compound for extinction. In this case, on the first extinction trial, the error term would approach -2 ($\sum V=V_A+V_B$) and we would therefore theoretically expect more extinction to occur for target cue A than if only A had been presented for extinction. But this is not a universal theoretical prediction. According to the Pearce configural model, presenting an AB compound for extinction in this simple procedure would not increase prediction error. This is because $\sum V$ in Pearce’s configural model is determined as a weighted sum of the associative strengths of all configurations known to the system with the weights being formed by the similarities between the configuration actually present (AB in this case) and all configurations in the system (A, B, and AB in this case). Assuming the similarity between each of the elements and the AB compound is $\frac{1}{2}$ (Pearce, 1994) and since $V_{AB}=0$ we would have $\lambda -\sum V\rightarrow -1$ which is the same as we would have if A was presented alone for extinction. Furthermore, since the associative change would occur to configuration AB the impact would only be on responding to the target cue A via generalisation, the associative strength of configuration A itself would remain unaffected.

In fact, there have been numerous demonstrations, especially in animal studies, which have shown that increased prediction error during extinction can result in more extinction (e.g. Rescorla, 2000, 2006), but see also Pearce and Wilson (1991) for an exception. In Rescorla (2000) rats were trained with two cues A and X as signals for food (A+ and X+ trials) and with a third cue B which was non-reinforced (B- trials). The animals were then divided into four groups with one group receiving extinction trials with an AX compound stimulus (AX- trials), and the other groups receiving extinction trials with X alone (X- trials), a BX compound (BX- trials), or no extinction trials at all. In a test presentation of X group AX- showed the least responding of all indicating that the AX- extinction trials had resulted in the most complete extinction. We term this procedure ‘super-extinction’ (as used in Hermans et al., 2006 and Jacoby and Abramowitz, 2016) after its mirror analogue with ‘super-conditioning’ (e.g. Williams & McDevitt, 2002) and distinguish it from a related procedure ‘deepened-extinction’. In super-conditioning, the acquisition of associative strength for a target cue is enhanced by reinforcement of that target in compound with an inhibitory cue, whereas in super-extinction, the extinction of associative strength for a target cue is enhanced by non-reinforcement of that target in compound with an excitatory cue.

Super-extinction differs from deepened-extinction in that deepened-extinction is a ‘post-extinction’ procedure (Leung et al., 2012) involving two extinction phases. In the first extinction phase of a deepened-extinction procedure, the target cue is extinguished alone and only after this initial extinction of the target is a compound involving the target and a second, non-extinguished, excitatory cue introduced. In the second extinction phase, this compound is presented non-reinforced. This difference could be theoretically as well as practically important since according to the Rescorla–Wagner model, in a simple super-extinction procedure, there should be more rapid extinction when compared to extinction of a single cue but asymptotically both procedures would lead to the associative strength of the target cue falling to zero. In contrast, in a deepened-extinction procedure, it would be possible for the target cue to acquire inhibitory strength and so cue-exposure treatment with deepened-extinction may be more effective than single cue or super-extinction e.g. because the target would be less likely to have residual post-treatment associative strength. And, at least in a simple case, the superiority of the deepened-extinction procedure is also anticipated by the Pearce configural model and by a frequently cited development of the Rescorla–Wagner model, the configural Rescorla–Wagner model (e.g. Rescorla, 1973) which will be described in more detail below.

In the current study, we had two primary objectives. First, we sought to examine further the extent to which extinction is impacted, in human participants, by super-extinction and deepened-extinction procedures. Second, we sought to examine which of the three related associative models, each based on error correction, would provide the best account of participant behaviour during our extinction procedures. In relation to the first point, as already mentioned, there are many studies showing that increased prediction error can increase extinction relative to simple single-cue extinction procedures. However, notably in human studies, there are several published investigations that indicate that strategies to increase prediction error do not always have the anticipated effect. For example, Griffiths et al. (2017), using a predictive learning task, found that whilst a super-extinction procedure resulted in faster extinction than single-cue extinction (Experiment 1: phase 2 A- trials versus phase 3 CD- trials) there was no evidence that single-cue extinction followed by compound extinction was more complete than extended single-cue extinction, in fact the reverse appeared to be the case (Experiment 2: A/B ‘v’ C/D ). And, a recent set of five experiments following up the work of Griffiths et al. whilst consistently finding evidence that super-extinction produced faster extinction than single-cue extinction (e.g. Experiment 1: phase 2 A- trials versus phase 3 CD-) trials) failed to show an effect of prediction error (e.g. Experiment 1: cue B versus D). Cues B and D had the same number of compound extinction trials but cue B was extinguished in compound with a cue that was already extinguished whilst cue D was extinguished in compound with an excitor.

In relation to the second point, it is clear a) that the simple Rescorla–Wagner model can predict more rapid extinction in super-extinction than in single-cue extinction and that asymptotically deepened-extinction will be more effective than super-extinction in the simplest procedures (see below). It is also clear b) that these predictions, whilst shared by the configural Rescorla–Wagner model, are only partially shared by the Pearce configural model. To elaborate, as described above, the Pearce configural model expects the same rate of extinction for a single cue A as for an AB compound but the configural Rescorla–Wagner also expects faster extinction of the compound because of the larger prediction error caused by the presence of two excitatory cues in the absence of reinforcement. In addition, c), both the configural Rescorla–Wagner model and the Pearce configural model anticipate response recovery in the super-extinction condition whereas the Rescorla–Wagner model does not. This is because the target cue A is actually extinguished during super-extinction in the Rescorla–Wagner model but in the configural Rescorla–Wagner model the configural cue in the AB compound becomes inhibitory, protecting the target from extinction. In the Pearce configural model the AB configuration becomes inhibitory during super-extinction but the configural unit for cue A is not affected and its associative strength (moderated by generalisation of inhibition from AB) is revealed in recovery test.

However, despite this, we do not necessarily have the basis for a theoretically decisive test because these model predictions are dependent on both procedural and model parameters. For example, as super-extinction will asymptotically be equivalent to single cue extinction if there are too many extinction trials then differences between single cue and super-extinction conditions may be harder to detect. And if the second stage of a deepened-extinction procedure is introduced too early, then any differences between super-extinction and deepened-extinction may also be difficult to detect. Furthermore, optimal procedural parameters will depend on model parameters and some caveats need to be attached to the discussion of model predictions in the previous paragraph. First, the discussion was based on the simplest procedures for studying super and deepened-extinction only involving A+ and B+ training during the initial acquisition phase, only involving AB- trials during super-extinction, and only involving A- followed by AB- trials during deepened-extinction. Introducing other cues (e.g. fillers and contexts) may have an impact on model predictions. Second, it has been argued that in the Pearce configural model, a test on target cue A after compound extinction will be equivalent to extinction of a single cue i.e. that the Pearce configural model does not predict more extinction following a compound extinction procedure (Urcelay et al., 2009). However, we carried out calculations to support the arguments made above (see Footnote 1 for link to ESM including spreadsheet showing these calculations.) but these are only valid under specific conditions and simulations done under different conditions may well produce different results.

Additionally, since the predictions outlined above are based on associative strength, without assuming any more than a monotonic mapping to response strength, they are qualitative rather than quantitative. Therefore, in what follows, we apply a SoftMax function (e.g. Ahn et al., 2008; Wikipedia, 2020; Yechiam & Busemeyer, 2005) to map between associative strength and response probability in order to estimate the likelihood of observed participant behaviour under maximum likelihood parameterisation of each of our three models. With these likelihood estimates, we use Akaike weight analysis (Burnham & Anderson, 2002; Wagenmakers & Farrell, 2004) to provide further evaluation of our three models by examining their capacity to model observed behaviour rather than make specific predictions.

We pursued our two primary objectives (comparing super and deepened-extinction to standard single-cue extinction and comparing the fitting capacity of three related associative models) by analysing data collected in a predictive learning experiment involving human participants. All participants underwent multiple experimental phases, including the acquisition and extinction of predictive responses, followed by a recovery test to assess the robustness of extinction to contextual change. Participants were tested in three groups differing in the type of extinction procedure used as detailed fully in what follows.

## Method

### Participants

A sample of 207 student participants was recruited through a subject pool run in the Department of Psychology at the University of Southampton, by posted adverts, and by word of mouth. Their average age was 19.5 years, 165 identified as female, 41 identified as male, one identified as non-binary, and one did not provide gender information.

### Learning task

The online procedures were approved by the Ethics Committee at the University of Southampton. On arrival at the experiment website, participants read an information sheet and provided informed consent before giving age and gender information and completing the learning task described below. Participants also completed some questionnaires and another behavioural task as part of a separate investigation. The procedures took around 45 min in total, and participants were awarded course credits on completion.

The learning task was programmed by the first author using jsPsych and run on a JATOS server hosted at the University of Southampton. The task was designed to have a ‘game-like’ appearance. Participants were presented with a background story which stated that they were part of a research team studying the eating habits of a friendly unidentified life form (FULF). The learning task consisted of a series of trials. On each trial, cues were presented on the screen (either one or two images of food items with the screen background forming the context for that trial), followed by FULF’s reaction, or lack of reaction, to that food item or food item combination. FULF’s reaction, the outcome, was one of three possibilities – happy, sad, or neutral (no change from a baseline state), as per the experimental design. The happy and sad outcomes were the reinforced outcomes and participants were instructed to press the ‘h’ or ‘s’ keys to predict these outcomes and to refrain from pressing any key if the neutral, no change (non-reinforced), reaction was expected. Three outcomes were used, corresponding to X, Y, and Z as detailed below, so that some reinforced trials (involving outcome Y) could be delivered during the extinction phase whilst cues paired with outcome X were being extinguished. Previous experience in this lab has indicated that if reinforcement stops completely during an extinction phase, then responding stops very quickly and could therefore obscure important differences between groups. Participants were instructed to respond while the food item was present, before seeing the reaction, in order to predict FULF’s reaction. The instructions also asked participants to try and maximize the number of correct predictions and minimise the number of incorrect predictions. The food items were present for 2 s, during which participants had to make their prediction for the trial. Any valid prediction responses during that period were recorded, otherwise a prediction for the neutral outcome was recorded. Next, the participants were shown the outcome for one and a half seconds, and finally, a fixation cross was presented for a further 2 s before the next trial started.^1^

### Design and procedure

The design of the learning task is given in Table 1. Each participant experienced a total of 179 trials split into five phases – 144 acquisition phase trials, 16 extinction phase 1 trials, 16 extinction phase 2 trials, and two test phases, summation test and recovery test. The participants were divided into three independent groups receiving different experimental treatments during the extinction phases. The summation test phase came first and consisted of two trials and the experiment finished with the recovery test phase which was a single trial. Acquisition took place in context A:, the extinction and summation phases took place in context B:, and recovery was in context C:. Cues were presented in trials that were either reinforced by presentation with an outcome or non-reinforced by presentation without an outcome. There were two types of reinforced trials, those with happy and those sad outcomes which are coded X and Y in Table 1; the non-reinforced trials are coded Z. The assignment of happy and sad outcomes to X and Y was randomised so that for approximately half of the participants X corresponded to sad and Y corresponded to happy and vice-versa for the other half. The acquisition and extinction phases had multiple trials divided into blocks with trial order randomised independently for each participant within block. The acquisition phase had four blocks. Within each acquisition block there were four presentations of each cue with outcomes delivered according to a continuously reinforced (e.g. four $C\rightarrow Y$) or partially reinforced schedule (e.g. three $A\rightarrow X$ and one $A\rightarrow Z$ trials as per the design in Table 1). The images used for each cue in the design were selected at random, without replacement from a set of ten, for each participant. Throughout the experiment cues and outcomes were presented in one of three visually distinctive contexts as per the design. Screen background images were used to provide context cues. For each participant backgrounds were selected at random, without replacement from a set of five backgrounds, to serve each of the three contextual functions (A:, B:, and C:).

Each of the two extinction phases contained eight blocks, with each block containing one trial of each of the types shown in Table 1. Cue A was the critical cue for testing the effects of deepened and super-extinction. For the control group cue A was extinguished alone during both extinction phases. In the deepened-extinction condition, A was extinguished alone during extinction 1 and in compound with cue B during extinction 2. In the super-extinction condition, cue A was extinguished in compound with B during both extinction 1 and extinction 2. Cue G was used in a summation test to assess the inhibitory strength of the extinction context after extinction was finished. Cue A was presented for a renewal test in a novel context, context C:, after the summation test.

It was assumed that if compound extinction was to increase extinction above that seen with single-cue extinction, participants would have to sum outcome expectations generated by multiple cues in the manner suggested by associative models, such as the Rescorla–Wagner model. To maximise the likelihood that such summation would occur cues A and B were partially reinforced with outcome X during the acquisition phase and cues K and L were partially reinforced with outcome Y. Cues K and L were also presented in a continuously reinforced KL compound as a ‘demonstration’ of cue additivity. Previous research with human participants has found that such additivity demonstrations encourage participants to sum outcome expectations generated by multiple cues (e.g. Lovibond et al., 2003).

**Table 1:** Design of the learning task

	Acquisition	Extinction 1	Extinction 2	Summation	Recovery
	A:	B:	B:	B:	C:
Super	A$\rightarrow$X x12	AB$\rightarrow$Z x8	AB$\rightarrow$Z x8	G$\rightarrow$Z x2	A$\rightarrow$Z x1
Extinction	A$\rightarrow$Z x4	C$\rightarrow$Y x8	C$\rightarrow$Y x8		
	B$\rightarrow$X x12				
	B$\rightarrow$Z x4				
	C$\rightarrow$Y x16				
	D$\rightarrow$Z x16				
	E$\rightarrow$Z x16				
	G$\rightarrow$X x16				
	K$\rightarrow$Y x12				
	K$\rightarrow$Z x4				
	L$\rightarrow$Y x12				
	L$\rightarrow$Z x4				
	KL$\rightarrow$Y x16				
Deepened	A$\rightarrow$X x12	A$\rightarrow$Z x8	AB$\rightarrow$Z x8	G$\rightarrow$Z x2	A$\rightarrow$Z x1
Extinction	A$\rightarrow$Z x4	C$\rightarrow$Y x8	C$\rightarrow$Y x8		
	B$\rightarrow$X x12				
	B$\rightarrow$Z x4				
	C$\rightarrow$Y x16				
	D$\rightarrow$Z x16				
	E$\rightarrow$Z x16				
	G$\rightarrow$X x16				
	K$\rightarrow$Y x12				
	K$\rightarrow$Z x4				
	L$\rightarrow$Y x12				
	L$\rightarrow$Z x4				
	KL$\rightarrow$Y x16				
Control	A$\rightarrow$X x12	A$\rightarrow$Z x8	A$\rightarrow$Z x8	G$\rightarrow$Z x2	A$\rightarrow$Z x1
	A$\rightarrow$Z x4	C$\rightarrow$Y x8	C$\rightarrow$Y x8		
	B$\rightarrow$X x12				
	B$\rightarrow$Z x4				
	C$\rightarrow$Y x16				
	D$\rightarrow$Z x16				
	E$\rightarrow$Z x16				
	G$\rightarrow$X x16				
	K$\rightarrow$Y x12				
	K$\rightarrow$Z x4				
	L$\rightarrow$Y x12				
	L$\rightarrow$Z x4				
	KL$\rightarrow$Y x16				

Characters before $\rightarrow$ give the cues for a trial type, characters after $\rightarrow$ give the outcome. ‘Z’ codes for non-reinforced trials, ‘X’ and ‘Y’ code for the two different types of reinforced trials that were used. The number of trials of each type are given e.g. $\times$4. The columns indicate successive phases of the experiment, from left to right, and the characters before colons indicate the context that is in force during each phase

Additional cues C, D and E were used to equate the number of different outcome types on the single-cue trials during acquisition. Cue C was presented with outcome Y during the extinction phase, as in the acquisition phase, to provide some continuity between phases to avoid giving the impression that all reinforcement stopped suddenly after the change from acquisition to extinction context.

### Data selection and analysis

All analyses were carried out in R (R Core Development Team, 2020). Thirty-three of the 207 participants were excluded due to poor performance during the acquisition phase, leaving 174 participants for the analyses reported below. Since our primary aim was to study extinction of responding to cue A we required that participants had actually acquired appropriate responding to cue A during the acquisition phase. For each participant, we constructed two binary vectors that were then compared using one-sided Wilcoxon rank-sum tests. The first vector had length 4 and was used to represent responses to cue A during the last four trials of its presentation in the acquisition phase – X responses were coded 1, with any other responses coded 0. The second vector had length 12 and was used to represent responses to cues C, D, and E during their last four presentations of the acquisition phase, again X responses were coded 1 with any other responses coded 0. Cues C, D, and E were never paired with outcome X during the acquisition phase (C was continuously reinforced with outcome Y, D and E were continuously non-reinforced), and A was paired with outcome X on 75% of its presentations. Therefore, participants were included if the cue A vector was significantly greater (p$$ <.05$$) than the cue CDE vector and excluded otherwise.

#### Effects of extinction procedures

We compared our three extinction procedures to determine whether or not there was any evidence for a) more rapid extinction using a compound of two excitatory cues as compared to extinction of a single excitatory cue and b) more complete extinction in deepened and super-extinction procedures as compared to a standard single-cue extinction procedure. In the case of a), we fitted a general linear mixed model with a binomial link function and random effect intercepts. The fixed effect terms were a between-subjects contrast for group, within-subjects contrasts for trial, and interaction contrasts for group and trial. The dependent variable was a binary valued vector indicating whether or not participants predicted outcome X on the last cue A trial of the acquisition phase and on each of the eight extinction 1 trials involving cue A. Participants for the control and deepened-extinction groups were treated as one ‘standard single-cue’ extinction group, dummy coded 0, for the purpose of this analysis since they were treated identically up until the end of extinction 1 phase. The combined groups were contrasted with the super-extinction group, dummy coded 1, which had extinction of an AB compound during the extinction 1 phase. Eight dummy-coded variables were used to contrast each of the extinction 1 phase trials with the last cue A trial of the acquisition phase.

In the case of b) a general linear model with a binomial link function was used to contrast the group responses in the recovery test phase. In addition, we also examined whether responding in the recovery test phase was associated with suppression of responding to cue G in the Summation test. According to the protection from extinction account of response recovery the extinction context becomes inhibitory and release from that inhibition causes recovery of responding. Additional Wilcoxon rank-sum and Kruskall–Wallis tests were therefore carried to compare the amount of responding in the Summation test for our three experimental groups and for those who did and did not respond during the recovery test phase.

We also investigated whether or not there would be summation effects on the introduction of stimulus compounds on the first trial of extinction 1 for the super-extinction group and on the first trial of extinction 2 for the deepened-extinction group. Summation effects would provide evidence of additivity, which is required for the error-correction mechanisms described in the models considered in the introduction to generate increased rates of learning during compound extinction. A Wilcoxon signed-rank test was used to assess the significance of the increase in the likelihood of an x-response between the last trial of extinction 1 and the first trial of extinction 2 in the deepened-extinction group.

#### Model evaluation

Three models were studied– the Rescorla–Wagner model, the configural Rescorla–Wagner model, and the Pearce configural model in each of three steps. First, maximum likelihood parameter estimates were obtained for each model and participant. Second, using these parameter estimates, simulations of the experimental design were carried out and the expected (model) responses generated by simulation were compared to the observed (participant) responses. Third, models were compared using Akaike weight analysis to determine the best model overall and in order to assess the best model for each participant (Burnham & Anderson, 2002; Cavagnaro et al., 2016; Farrell & Lewandowsky, 2018; Wagenmakers & Farrell, 2004).

##### The Rescorla–Wagner model

The canonical form of the Rescorla–Wagner model is given in Eq. 2 (Rescorla & Wagner, 1972). In Eq. 2$\Delta V_{ijk}$ is the change in the associative strength (*V*) that occurs on trial *i* between cue *j* e.g. one of the foods eaten by the FULF on that trial (labelled $A \dots E, G, K, L$ in Table 1) and outcome of that trial. $\Delta V$ is a function of two learning rate parameters, $\alpha$ a learning rate for cues and $\beta$ a learning rate for outcomes, and the parenthesised error term. In the error term $\lambda _k$ represents the outcome of the trial and takes the value of 1 or 0 for the occurrence and non-occurrence of an outcome, respectively. $\Sigma V_{ijk}$ is the associative strength for outcome *k* summed over the *n* cues present on the trial.

2 $$\begin{aligned} \Delta V_{ijk}=\alpha \beta (\lambda _k-\sum _{j=1}^{n} V_{ijk}) \end{aligned}$$

The Rescorla–Wagner model was implemented with two values of $\alpha$, $\alpha _{ctx}$ and $\alpha _{cue}$, to allow different learning rates for different categories of cues. We allowed the diffuse context cues provided by the screen background that were stable within different phases of the experiment to have a different learning rate than the discrete food cues, which changed from trial to trial. We also allowed for two values of $\beta$, $\beta _{us}$ and $\beta _{\sim us}$, to allow for the possibility that the learning rate may differ on reinforced and non-reinforced trials.

##### The configural Rescorla–Wagner model

The configural Rescorla–Wagner model was implemented in the same way as Eq. 2 except an additional class of cue was introduced to represent stimulus configurations. In the Rescorla–Wagner model cues are considered ‘standalone’ elements representing the intrinsic physical properties of a stimulus. However, this is generally believed to be an oversimplification with evidence indicating that configural cues can be produced when multiple stimuli occur together (e.g. Rescorla, 1973; Wagner & Rescorla, 1972; Woodbury, 1943). In our implementation of the configural Rescorla–Wagner model we coded a unique configural cue to represent each pairwise cue combination. For example the cues on a trial involving presentation of cue *A* in context $A\!:$ would be encoded for simulation with three cues *aAw* where *a* is context $A\!:$, *A* is cue *A*, and *w* is the configural cue generated by the conjunction of *A* and $A\!:$. For an *AB* compound presented in context $B\!:$ the encoding would involve six cues *bABxyz*, *b* for context $B\!:$, *A* for cue *A*, and *B* for cue *B*, plus configural cues *x*, *y*, and *z* which represent the pairwise cue combinations as follows: $bA\rightarrow x$, $bB \rightarrow y$, and $AB \rightarrow z$. The configural Rescorla–Wagner model therefore has one more parameter than the Rescorla–Wagner model, an additional learning rate parameter $\alpha _{cfg}$ allowing different learning rates now for three categories of cue (context cues, discrete cues, and configural cues).

##### The Pearce configural model

Pearce (1994) developed a widely cited configural model of associative learning which, despite the common moniker ‘configural’, operates on quite different principles than the configural Rescorla–Wagner model. The main difference between these models is in the way in which the cues are processed. In the Rescorla–Wagner model and the configural Rescorla–Wagner model each cue enters into individual associations with the outcomes. In contrast, in the Pearce configural model, cues are grouped into configurations, and a configuration is formed by each unique pattern of cues encountered during learning and the configurations, rather than cues, are the units which enter into associations with the outcomes. For example, referring again to design Table 1, during the acquisition phase a configural unit *aA* would be used to represent the stimulus pattern when cue *A* was encountered in context $A\!:$ and in the extinction phase a configural unit *bAB* would represent the cue compound *AB* presented in context $B\!:$.

In Eq. 3$\Delta c_{ik}$ is the change in the associative strength between the configuration present on that trial ($c_i$) and the trial outcome. Equation 3 is of the same form as the Rescorla–Wagner model but the error term is computed as the difference between $\lambda _k$ and a weighted sum of the associative strengths of all the stimulus configurations known to the system. The weights are provided by the similarities between $c_i$ and each of the *n* configurations in the system with the similarity between any two configurations *a* and *b* given as a function of the number of cues common to both configurations, $n_{ab}$, and the number of cues in each configuration, $n_a$ and $n_b$, as shown in Eq. 4. In Eq. 4*d* is a discrimination sensitivity parameter with larger values reducing the similarity and therefore increasing discrimination between configurations (Kinder & Lachnit, 2003).

3 $$\begin{aligned} \Delta V_{c_{ik}}=\alpha \beta (\lambda _k-\sum _{j=1}^{n} S\left( c_i,c_j\right) V_{c_{jk}}) \end{aligned}$$

4 $$\begin{aligned} S(a,b)= \left( \frac{n_{ab}}{\sqrt{n_a}\sqrt{n_b}}\right) ^d \end{aligned}$$

##### Parameter estimation

The maximum likelihood parameters were estimated using R code written by the last author (see Footnote 1 for availability) which was run in R version 4.0.3 using Nelder-Mead optimisation via package optimx version 2022-4.30 (Nash & Varadhan, 2011; R Core Development Team, 2020). The optimisations found, for each participant and model, a parameter vector for that model, $\varvec{\theta }$, which minimised $\mathcal {L}$ over the n=179 trials of the experiment as shown in Eq. 5:

5 $$\begin{aligned} \mathcal {L}=-\sum \limits _{i=1}^n \ln P(R_i) \end{aligned}$$

The models used one-step lookahead, making probabilistic predictions for each available response (X, Y, and Z) on trial *n* on the basis of what had been learned up to and including trial $n-1$. $P(R_i)$ was the model probability for the observed response on trial *i*. Three possible responses were available to participants on each trial – they could predict outcome X, outcome Y, or outcome Z and $P(R_i)$ was a SoftMax function of the associative strengths of the cues present on trial *i* and a sensitivity parameter *g* as shown in Eq. 6 (cf. Ahn et al., 2008; Wikipedia, 2020; Yechiam & Busemeyer, 2005).

6 $$\begin{aligned} P(R_i)=\frac{\exp (gV_{ir})}{\sum \limits _{k=x}^z\exp (gV_{ik})} \end{aligned}$$

$V_{ir}$ in the numerator or Eq. 6 is the associative strength for the outcome corresponding to the observed response summed over all cues present on the trial and the denominator includes the associative strength summed over all outcomes and all cues present on the trial. When $g\rightarrow 0$ Eq. 6 results in guessing behaviour with the response probabilities approaching $\frac{1}{n}$ where *n* is the number of response options ($n=3$ in this case). When $g\rightarrow \inf$ Eq. 6 results in maximisation with the probability of the response for which the associative strength of the cues present on that trial is highest approaching 1.

**Fig. 1:**
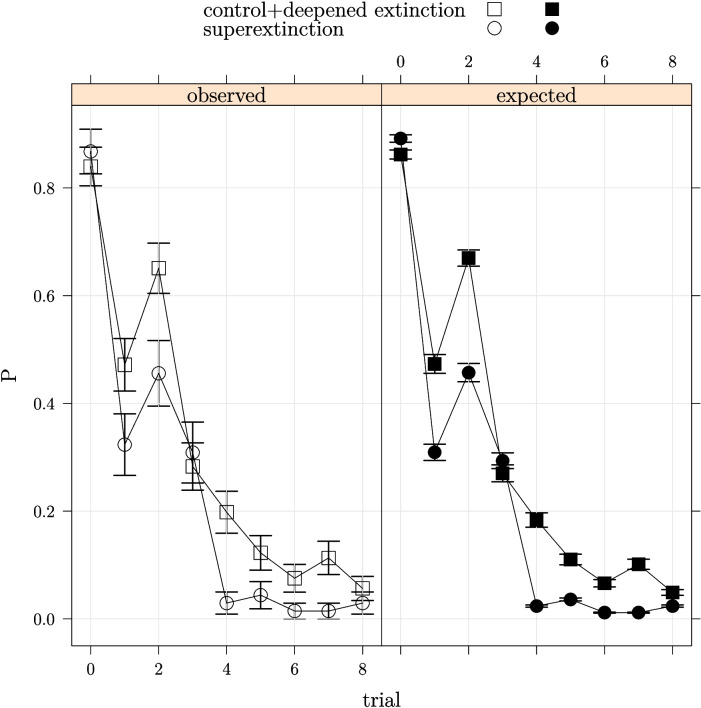
Average proportion of x-responses observed and expected (± 1 s.e.) from the general linear mixed model used to test for differences in rate of extinction between the super-extinction and the combined control and deepened-extinction groups. The first point in each panel is end of acquisition and the remaining eight points are extinction 1 phase trials

The optimisations included some constraints on the parameter values to provide numerical stability and to preserve the psychological sense of the parameters in the current modelling context (e.g. although some analyses have suggested a modification of the Rescorla–Wagner model which allows negative learning rates (e.g. Dickinson & Burke, 1996; Van Hamme & Wasserman, 1994) these were not used here). All learning rates were constrained to the range $[0.0001 \dots 0.75]$, *g* was constrained to the range $[0.0001 \dots 15]$, and *d* was constrained to the range $[0.0001 \dots 20]$. In addition, all optimisations were run with three initial values of $\varvec{\theta }$. One value came from an initial exploratory optimisation, and one value consisted of all parameters set to 0.1 except for *g*, which was set to 2, and the third initial value vector was set to a selection of random values. Finally, on completion of the optimisations, a sensitivity analysis was carried out to assess the importance of each parameter for each model. The details and full results of this analysis are presented in an extra-supplementary material paper (see Footnote 1 for availability), with the key results summarised below.

**Table 2:** Fixed effects from general linear mixed effects model for extinction 1 phase

	Estimate	Std. Error	z value	Pr($>$ $|$z|)	sig
Intercept	2.03	0.31	6.53	0.00	***
Group	0.18	0.50	0.37	0.71	
Trial1	−2.15	0.37	−5.88	0.00	***
Trial2	−1.23	0.36	−3.37	0.00	***
Trial3	−3.18	0.39	−8.17	0.00	***
Trial4	−3.75	0.41	−9.08	0.00	***
Trial5	−4.42	0.46	−9.70	0.00	***
Trial6	−5.04	0.51	−9.79	0.00	***
Trial7	−4.53	0.46	−9.75	0.00	***
Trial8	−5.38	0.56	−9.64	0.00	***
Group:Trial1	−0.96	0.60	−1.60	0.11	
Group:Trial2	−1.20	0.59	−2.03	0.04	*
Group:Trial3	−0.01	0.61	−0.02	0.98	
Group:Trial4	−2.38	0.92	−2.60	0.01	**
Group:Trial5	−1.27	0.84	−1.52	0.13	
Group:Trial6	−1.81	1.19	−1.52	0.13	
Group:Trial7	−2.32	1.17	−1.98	0.05	*
Group:Trial8	−0.75	0.98	−0.76	0.45	

Groups control and deepened-extinction contrasted with group super-extinction. Trial contrasts against end of acquisition. *** p < .001, ** p < .01, * p < .05

**Fig. 2:**
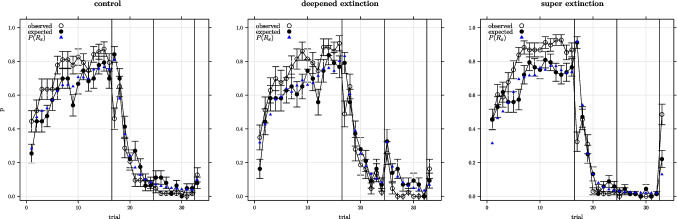
Average proportion of x-responses observed and expected for the Rescorla–Wagner model using maximum likelihood parameters on trials involving cue A by experimental condition (± 1 s.e.). *Vertical lines* separate acquisition, extinction 1, extinction 2, and recovery test phases. $P(R_x)$ is the average probability of an x-response used to parameterise the binomial distribution for generating random deviates for the model responses

## Results

### Effects of extinction procedures

Figure 1 shows that there is some evidence that the super-extinction group extinguished more rapidly across the extinction 1 phase trials although there is no indication of a summation effect on the first extinction trial when the super-extinction participants encounter AB compound cue for the first time. In fact, all groups show a marked reduction in responding on the first extinction trial. By the fourth extinction trial responding in the super-extinction group was markedly more suppressed than in the combined control and deepened-extinction group but thereafter responding equates by the end of extinction 1. Table 2 gives the fixed effect results from the general linear mixed effects model used to examine the extinction 1 phase data. Overall, the Group $\times$ Block interaction was significant with a likelihood ratio test comparing models with and without the interaction contrasts yielding $\chi ^2=$18.501 (df = 8, $p < .05$). Confirming visual impressions, the interaction contrast for the fourth extinction trial was significant ($p<.01$).

Figure 2 shows the observed x-responses on trials containing cue A by group across the course of the experiment. It can be seen that responding had stopped entirely by the end of the extinction 2 phase. In fact, only two participants responded on the last extinction 2 trial, one from the control group and one from the super-extinction group. However, this does not indicate extinction was complete – response recovery was observed when cue A was presented for test in context C. Wilcoxon signed rank tests comparing responding in the last extinction 2 trial with responding in the recovery test phase were significant for all groups (V = 5 , $p <.05$, V = 0 , $p <.05$, V = 17.5 , $p <.001$, for the control, deepened, and super-extinction groups respectively). However, the recovery effect was much stronger in the super-extinction group than in the other groups with the general linear model used to analyse these data showing that the deepened extinction group did not differ from the control group ($z=0.518$, *p* = .6) whereas the superextinction group did (*z* = 4.158, $p <.001$).

**Table 3:** Mean maximum likelihood parameters and $\mathcal {L}$ for Rescorla–Wagner model (standard error)

group	$\mathcal {L}$	$\alpha _{ctx}$	$\alpha _{cue}$	$\beta _{us}$	$\beta _{\sim us}$	*g*
c	93.232	0.214	0.463	0.415	0.299	6.051
	(4.6)	(0.024)	(0.026)	(0.026)	(0.028)	(0.398)
de	95.065	0.248	0.492	0.336	0.319	6.05
	(4.289)	(0.035)	(0.026)	(0.028)	(0.034)	(0.449)
se	88.324	0.132	0.521	0.365	0.426	5.231
	(3.075)	(0.019)	(0.02)	(0.023)	(0.026)	(0.301)
all	91.767	0.19	0.493	0.376	0.354	5.73
	(2.308)	(0.015)	(0.014)	(0.015)	(0.017)	(0.217)

**Table 4:** Mean maximum likelihood parameters and $\mathcal {L}$ for configural Rescorla–Wagner model (standard error)

group	$\mathcal {L}$	$\alpha _{ctx}$	$\alpha _{cue}$	$\beta _{us}$	$\beta _{\sim us}$	$\alpha _{cfg}$	*g*
c	90.655	0.226	0.254	0.287	0.204	0.277	7.014
	(4.634)	(0.026)	(0.023)	(0.025)	(0.025)	(0.029)	(0.382)
de	92.519	0.21	0.292	0.305	0.224	0.272	6.64
	(4.398)	(0.035)	(0.03)	(0.034)	(0.034)	(0.032)	(0.483)
se	84.323	0.128	0.227	0.261	0.262	0.381	6.787
	(3.189)	(0.017)	(0.019)	(0.022)	(0.03)	(0.027)	(0.349)
all	88.641	0.184	0.253	0.281	0.231	0.316	6.833
	(2.357)	(0.015)	(0.013)	(0.015)	(0.017)	(0.017)	(0.227)

There was no indication of a summation effect on the first trial of extinction 1 in the super-extinction group. In fact, x-responses markedly declined on introduction of the AB compound on the first trial of extinction 1 for this group. In contrast, there was a clear summation effect for the deepened-extinction group. The number of x-responses increased substantially between the end of extinction 1 and the first trial of extinction 2 on introduction of the AB compound ($p <.01$) as confirmed in a Wilcoxon signed rank test. See Fig. 2.

Finally, the average number of x-responses (minimum = 0, maximum = 2) to cue G in the Summation test was actually lower (0.409) in those who did not respond in the recovery test than it was for those who did respond in the recovery test (0.49), but the differences were not significant (W = 2598.5, *p* = .08). This suggests that increased context inhibition, which would have reduced responding in the Summation test, was not linked to greater responding in the recovery test. Supporting this, a Kruskal–Wallis test comparing the number of x-responses in the Summation test for the three groups produced $\chi ^2=$4.779 (df = 2, *p* = .09) – the experimental groups did not differ in the Summation test phase.

### Model evaluation

Average maximum likelihood parameter estimates and $\mathcal {L}$ values are shown in Tables 3, 4, and 5 for the Rescorla–Wagner model, the configural Rescorla–Wagner model, and the Pearce configural model respectively for each experimental condition and overall. As can be seen in Tables 3–5 the average $\mathcal {L}$ values were in the range of 84 to 95 indicating that the average model probabilities for the observed responses were in the range of 0.62 to 0.58 and the model fits tended to be better (lower $\mathcal {L}$) for the super-extinction group and worst for the Rescorla–Wagner model but note that these overall model assessments do not take into account model complexity which is done in Section Akaike weight analysis below.

#### Simulations

**Fig. 3:**
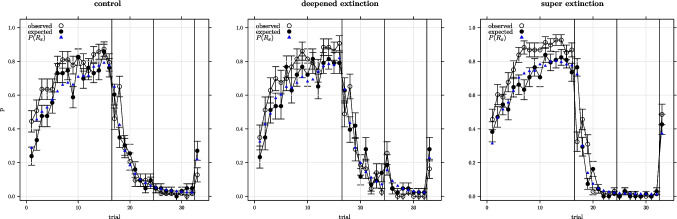
Average proportion of x-responses observed and expected for the configural Rescorla–Wagner model using maximum likelihood parameters on trials involving cue A by experimental condition (± 1 s.e.). *Vertical lines* separate acquisition, extinction 1, extinction 2, and recovery test phases. $P(R_x)$ is the average probability of an x-response used to parameterise the binomial distribution for generating random deviates for the model responses

**Fig. 4:**
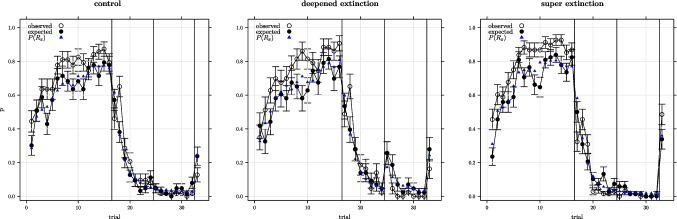
Average proportion of x-responses observed and expected for the Pearce configural model using maximum likelihood parameters on trials involving cue A by experimental condition (± 1 s.e.). *Vertical lines* separate acquisition, extinction 1, extinction 2, and recovery test phases. $P(R_x)$ is the average probability of an x-response used to parameterise the binomial distribution for generating random deviates for the model responses

Simulations of the experimental design shown in Table 1 were carried out for each model and participant using maximum likelihood parameters. Figures 2, 3, and 4 show the observed responses for each experimental condition and model alongside the model-predicted responses. Data are shown for trials with cue A present and for outcome X responses. Participant responses were coded 1 if an outcome X response was observed and 0 otherwise and the plotted data is averaged across participants. The model predicted responses were generated from random Bernoulli deviates obtained for each trial and participant (1 coding the model predicting an X response and 0 otherwise) with the distribution for each trial parameterised by $P(R_x)$ for that trial with plotted data showing the model predicted responses averaged across participants. The figures also show the average Bernoulli parameters used to obtain the deviates. As can be seen, the models generally provide a good fit to the observed data with major exceptions being in the case of the Rescorla–Wagner model where there is a summation prediction on the first extinction trial (none was seen) and where the predicted recovery on the last trial was substantially lower than that observed.

#### Akaike weight analysis

Table 6 provides the results of overall Akaike weight analyses and shows that the Pearce configural model performed best. Each of the models discussed above was evaluated in addition to a simple baseline guessing model in which it was assumed that for all trials and participants $P(R_x)=P(R_y)=P(R_z)=\frac{1}{3}$. We used the finite sample correction form of Akaike’s information criterion ($AIC_c$) as given in Eq. 7. In Eq. 7*V* is the number of parameters and *n* is the number of data points over which $\mathcal {L}$ was computed.

7 $$\begin{aligned} AIC_c= 2 \mathcal {L} + 2V + \frac{2V(V+1)}{n-V-1} \end{aligned}$$

**Table 5:** Mean maximum likelihood parameters and $\mathcal {L}$ for Pearce configural model (standard error)

group	$\mathcal {L}$	$\alpha _{pat}$	$\beta _{us}$	$\beta _{\sim us}$	*d*	*g*
c	91.159	0.531	0.482	0.309	2.069	6.933
	(4.522)	(0.024)	(0.03)	(0.033)	(0.15)	(0.469)
de	93.16	0.578	0.427	0.261	2.564	6.391
	(4.433)	(0.028)	(0.032)	(0.034)	(0.456)	(0.513)
se	83.962	0.553	0.482	0.316	2.57	6.111
	(3.237)	(0.021)	(0.026)	(0.029)	(0.16)	(0.369)
all	88.841	0.551	0.468	0.3	2.387	6.478
	(2.347)	(0.014)	(0.017)	(0.018)	(0.14)	(0.256)

The best model has the lowest $AIC_c$ value, and the column $\Delta AIC_c$ in Table 6 provides the $AIC_c$ difference between the best model, the Pearce configural model, and each model listed. $\Delta AIC_c > 10$ indicates that a model has ‘essentially no support’ in the context of the current data and competing models (Burnham & Anderson, 2002). The probability of each model being the best model in the context of the current data and competing models is given by the Akaike weights ($wAIC_c$) in Table 6 computed as in Eq. 8. In Eq. 8 the $\Delta AIC_c$ value for each model *i* is normalised by dividing by the $\Delta AIC_c$ values summed over the *K* models.

8 $$\begin{aligned} w_iAIC_c=\frac{exp\left( \frac{-1}{2} \Delta _iAIC_c\right) }{\sum \limits _{k=1}^K exp\left( \frac{-1}{2} \Delta _kAIC_c\right) } \end{aligned}$$

The average $\mathcal {L}$ was actually slightly smaller for the configural Rescorla–Wagner model than for the Pearce configural model, but the configural Rescorla–Wagner model was not the best model overall due to the Akaike parameter penalty. Although the Pearce configural model was the best model overall, it was not the best model for every individual. $\Delta AIC_c$ and $wAIC_c$ values were computed for each participant and it was found that the Pearce configural model was the best model in 107 cases, with 40 and 27 cases best fit by the configural Rescorla–Wagner model and by the Rescorla–Wagner model, respectively. In the extra-supplementary material (see Footnote 1), we follow up on these individual differences by classifying participants into those who were best described by each of the models and plotting the observed responses to cue A throughout the experiment, as shown in Figs. 2–4, but split according to group defined by ‘best-model’. Of interest is the fact that in a simplified experimental design the Pearce configural model predicted recovery effects in the super-extinction but not in the deepened-extinction conditions and the fact that this pattern was only shown in the Pearce-configural participants.

We directly follow Cavagnaro et al. (2016) to assess the evidence that each of the models could be the best model for all participants and find, in keeping with the foregoing, that the Pearce configural model is the most likely to be the best model for any randomly selected participant. The individual Akaike weights give the probability that each model is best for that individual and therefore the product of the weights across participants gives the joint probability that a model is best for all participants. In addition, the ratio of two Akaike weights provides the weight of evidence in favour (or against) of one model versus another. Putting this together Cavagnaro et al. (2016) define the group Akaike information criterion (*gAIC*) for model *i* as in Eq. 9. In Eq. 9 the denominator is *wAIC* for the guessing model so the $gAIC_i$ is the weight of evidence in favour of model *i* being best for all participants ($j=1\dots n$) in comparison to the guessing model.

9 $$\begin{aligned} gAIC_i=\prod \limits _{j=1}^n \frac{wAIC_{ij}}{wAIC{0j}} \end{aligned}$$

**Table 6:** Overall Akaike weight analyses using corrected AIC

Model	Parameters	$2\mathcal {L}$	$AIC_c$	$\Delta AIC_c$	$wAIC_c$
guessing	0	68434.8	68434.8	35728.2	< 0.000001
Rescorla–Wagner	5	31903.3	33724.9	1018.3	< 0.000001
configural Rescorla–Wagner	6	30801.4	33007.7	301	< 0.000001
configural model	5	30895.8	32706.6	0	$\rightarrow$ 1

The column ‘Parameters’ gives the number of parameters estimated for each participant for each model. There were 174 participants; therefore, for example, the number of parameters estimated for $\mathcal {L}_{Rescorla-W\,\,agner}$ was $5 \times 174=870$. $\mathcal {L}$ computed over 179 trials for each of 174 participants – i.e. over 31,146 data points

Furthermore, the Akaike weights can be used to parameterise a Dirichlet distribution with a parameter for each of the *i* models computed from Eq. 10.

10 $$\begin{aligned} \alpha _i=1 + \sum \limits _{j=1}^n wAIC_{ij} \end{aligned}$$

Once the distribution parameters are calculated, the probability that model *i* will be the best for a randomly chosen participant is given by Eq. 11. In Eq. 11 we sum over the *m* models to normalise $\alpha _i$.

11 $$\begin{aligned} P(best_i)=\alpha _i\left( \sum \limits _{i=1}^m \alpha _i\right) ^{-1} \end{aligned}$$

Table 7 provides the results of the analyses described above and shows that the Pearce configural model is twice as likely (p = 0.554) to be the best model for a randomly selected participant than the next best model (the configural Rescorla–Wagner model, p = 0.28).

#### Sensitivity analysis

One-parameter-at-a-time sensitivity analyses were used to assess the importance of the parameters in each model. Small changes were made to each parameter around their maximum likelihood values and relative changes in the likelihood values were computed (c.f. Saltelli et al., 2004; Wikipedia, 2024). In all cases, the relative change values were small (relative changes generally $<1\%$ for changes of up to $5\%$ in parameter values). And, summarising for parameters common across the models, in each case: *g* was the most important parameter, $\beta _{us}$ was more important than $\beta _{\sim us}$, and $\beta _{\sim us}$ was least or equal least important.

## Discussion

In the current experiment, we looked for differences between three different extinction procedures. In each case, ABC designs were used such that acquisition was carried out in one context (A:), extinction was carried out in a second context (B:), and a response recovery test was carried out in a third context (C:). In one condition, a standard single-cue extinction procedure was used, in another, super-extinction was used, and in a third condition, deepened extinction was used. Our first primary objective was to determine whether or not super and/or deepened extinction would produce more complete extinction than standard single-cue extinction. Although there was evidence that super-extinction produced faster extinction than single-cue extinction, as has been observed previously, we did not see this translated into any evidence of reduced recovery. In fact, we observed the opposite: the cue treated with super-extinction produced more responding in the recovery test. There was also no evidence that the deepened-extinction treatment resulted in reduced recovery as compared to the other groups, except possibly in the case of the participants with behaviour best described by the Pearce configural model as found in a *post hoc* analysis reported in the supplementary materials (see Footnote 1). In relation to our modelling exercise, we found that the Pearce configural model provided the best overall fit to the data after correcting for model complexity using Akaike weight analysis.

The evidence that super-extinction and/or deepened-extinction can result in more robust extinction is divided in the current literature. Whereas animal studies (e.g. Leung et al., 2012; Rescorla, 2000, for deepened and super-extinction, respectively) have shown that compound extinction can be more robust than cue alone extinction these results have not been established in human studies. Both Culver et al. (2015) and Coelho et al. (2015) found that deepened-extinction reduced spontaneous recovery of skin conductance responses to aversively trained and then extinguished CSs. In contrast, as already noted in the introduction, Griffiths et al. (2017) reported that super-extinction resulted in faster extinction and found that compound extinction led to less extinction than single-cue extinction. Other human studies have also found effects opposite to those expected. Lovibond et al. (2000) showed reduced extinction in the presence of an inhibitor (protection-from-extinction) and in the presence of an excitor. Similar results were also reported by Griffiths and Westbrook (2012) and Holmes et al. (2014). The current result adds to those and represents something of a paradox – how can the presence of multiple excitatory cues, as used in super-extinction, result in apparently faster extinction and greater response recovery?

**Table 7:** Comparison of models on group *AIC* and probability of each model being the best model for a randomly chosen participant

Model	$\log gAIC$	P(best)
guessing	0	0.006
Rescorla–Wagner	17349.8	0.16
configural Rescorla–Wagner	17707.3	0.28
Pearce configural model	17858.9	0.554

Faster extinction can be explained in terms of the greater prediction error generated by presentation of excitatory compounds and we have some evidence for additivity effects in the summation effect that was seen at the start of extinction 2 in the deepened extinction group. A similar effect was expected at the start of extinction 1 but not observed despite our design including an additivity demonstration that should have helped to facilitate (e.g. De Houwer et al., 2002; Lovibond et al., 2003). However, quite possibly the novelty of entering context B: at the start of extinction 1 could have served to mask that effect. So, given that additivity is driving faster extinction, what then leads to greater response recovery?

One possibility is that the extinction context acquires more inhibitory strength in the super-extinction condition than in the other conditions, and this could cause protection from extinction. However, we found no evidence for greater context inhibition in the super-extinction condition in our Summation test. However, in the case of the configural Rescorla–Wagner model there are additional configural cues that would acquire inhibitory strength during extinction and these differentiate the control, deepened, and super-extinction treatments; the effect of these cues would not be detected in the Summation test. For the control and deepened-extinction conditions during Extinction 1 our configural Rescorla–Wagner model coded the A$\rightarrow$Z trials with a cue B: for the context, cue A, and a third configural cue *w* representing the coincidence of B: and A. Both B: and *w* would become inhibitory and protect A from extinction. However, in the case of super-extinction, there are two additional configural cues – *x* for the coincidence of B: and cue B, and *y* for the coincidence of cues A and B (see Introduction for additional explanation). The result of this is that, according to the configural Rescorla–Wagner model, the super-extinction condition will result in more protection from extinction than the control and deepened-extinction conditions. The Pearce configural model can also predict more recovery in the super-extinction than in the control and deepened-extinction conditions. For the Pearce model, responding in the recovery test is based on generalisation between a novel, and hence associatively neutral, C:A configuration and configurations A:A, B:A, & B:AB.

Generalisation between C:A and excitatory A:A is equivalent in each condition but generalisation between C:A and inhibitory B:A & B:AB differs between groups. In the super-extinction condition B:AB is the only inhibitory configuration and there is less generalisation between B:AB and C:A than between B:A and C:A so generalised inhibition has the least impact in the super-extinction condition, hence most response recovery is seen.

Although the simple Rescorla–Wagner model has provided a hugely important stimulus to enquiries into the associative basis of human learning over many years (e.g. Le Pelley & McLaren, 2003; Miller et al., 1995; Shanks & Dickinson, 1987) it has long been acknowledged that it is inadequate in a number of respects, for example it cannot provide an account of learning that seems to require some kind of configural stimulus representation, such as negative-patterning or bi-conditional discrimination learning (e.g. Glautier et al., 2016; Pearce, 1994; Shanks et al., 1998). With this background, the results of studies of multiple-cue extinction procedures cited above, and the results and analyses of the current investigation we conclude that super-extinction, as understood in simple Rescorla–Wagner terms, may well indeed result in faster extinction but there is a risk that any apparent therapeutic benefit would not materialise due to an increased risk of response-recovery, which is not anticipated by simple Rescorla–Wagner. Furthermore, we were unable to find any benefit of deepened extinction over simple single-cue extinction.

As well as making a comparison between different extinction procedures, the current investigation sought to provide a test of three associative learning models in accounting for the behaviour observed across our three experimental conditions. Two of these models were ‘configural’ and these configural models outperformed the simple ‘elemental’ Rescorla–Wagner model. Furthermore, the Pearce configural model outperformed the configural Rescorla–Wagner model when model complexity was taken into account using Akaike’s information criterion. It is important to take model complexity into account during model evaluation because, in general, more complex models tend to produce better fits to the data at hand but they do not generalise as well to new data sets (e.g. Myung, 2000). To carry out this model evaluation, however, it was necessary to extend each model to map from associative strength to response probability. In some situations, there are qualitatively different predictions from associative models – e.g. adding a common feature to a feature negative discrimination (A+/AB- trials versus AC+/ABC- trials) is a more difficult discrimination for the Pearce configural model but an easier discrimination for the Rescorla–Wagner model (cf. Pearce, 1994; Pearce & Redhead, 1993; Thorwart et al., 2010). However, in the current paper, we did not have differential qualitative predictions available to distinguish between all three models but employing Eq. 6 to map from associative strength to response probability allowed us to compare the models on their fitting capacity.

All models provided generally good fits to the observed data (Figs. 2 ...4). The averaged minimised $\mathcal {L}$ ranging between $\approx$ 93 and 84 (Tables 3 ...5) which with 179 trials equates to average model probability for the observed response on each trial ranging between 0.59 and 0.62. Overall the Pearce configural model was a clear winner in this model fitting exercise. Unsurprisingly, the Pearce configural model was also the winner at the level of individual participant fits. The probability of the Pearce configural being the best model for a randomly chosen participant was 0.55 – in comparison, for the second-best model, the configural Rescorla–Wagner model, the probability of being the best model for a randomly chosen participant was 0.28.

Until now, we have focused on trying to explain the pattern of results in terms of three formal associative models, but it is appropriate to consider an alternative view about the underlying mechanisms for recovery effects. Rosas and Callejas-Aguilera (2006), see also Bouton (1997) and Nelson (2002), argued that during ABC/ABA recovery experiments, the participants will pay attention to and encode the context during the extinction phase so that extinction effects will become context dependent. Their explanation is based upon the idea that when there is a prediction error, it increases context processing. In relation to the current studies, the prediction error is maximal in the super-extinction condition and therefore extinction in this condition would become more context dependent than in the other conditions. This is an appealing explanation but speculative in the case of this specific study because we did not measure attention. However, recent work by Nelson et al. (2022) suggests a way to take this idea forward in future studies. Using eye-tracking apparatus, Nelson et al. classified their participants into sign or goal trackers based on the location (CS or US location, respectively) of their visual attention during learning. Subsequently, they found that a context shift reduced latent inhibition in sign-trackers, whereas the opposite was found in goal-trackers.

Before concluding, we consider two design issues that may serve to limit the inferences that can be drawn from this study. First, we relied on the recovery test to assess the associative impact of our extinction procedures on the target cue A. We chose to use an ABC design for this purpose because context C would be associatively neutral at the time of the test. One possible drawback of that design decision is that the novelty of the context may have impacted the sensitivity of this recovery test – and we saw some indication that novelty effects could mask summation effects on the first trial of extinction 1. However, in the event, we saw clear group differences on this test where we would expect any novelty effects to be equated across groups.

Second, and possibly more consequential, relates to the use of single-cue extinction in the control condition and during extinction 1 for the deepened-extinction group. This raises the possibility that any difference between AB compound extinction and single-cue A extinction is not mediated by the associative strength of B and, in the case of the control group versus the super and deepened-extinction groups, there is a difference in generalisation decrement between the end of extinction 2 and the recovery test. In this experiment, therefore, we have to acknowledge that the enhanced reduction in responding during extinction 1 may not be an associative effect, but we note that in many reported studies, additional controls are added in later experiments, which confirm that initial results are not due to some non-associative effects. For example in Rescorla’s (2006) study the initial experiments contrasted single and compound cue extinction (e.g. Experiment 1: A+, X+ acquisition; A-, X- extinction 1; followed by AX- or X- extinction 2) and additional controls confirming the specific associative effect of the compound extinction were added in Experiment 5 (see also Leung et al., 2012, Experiment 1 ‘v’ Experiment 2 for another example). With respect to the generalisation decrement between the end of extinction 2 and the recovery test it is true that using an AD- control condition would have ensured matched similarity between the stimuli presented at the end of extinction 2 and the test across groups but our objectives included practical as well as theoretical considerations. From the results of the experiment as conducted we can say that there is no evidence for an advantage in presenting cue compounds over single cues as is often done in cue-exposure treatments (e.g. Drummond & Glautier, 1994; Shiban et al., 2015) and, importantly, there is some evidence that compound extinction may actually worsen recovery and this is an important point. In summary, we found no evidence that compound extinction either by super-extinction or by deepened-extinction provided any advantage in terms of reduced response recovery than simple single-cue extinction. In fact, super-extinction actually increased response recovery and increased the initial rate of extinction. However, we did not find any evidence that a protection-from-extinction mechanism played a part in enhanced response recovery. We also found that our two configural models performed better than the simple Rescorla–Wagner model but the Pearce configural model in turn outperformed the configural Rescorla–Wagner model. Sensitivity analyses indicated that for all models, the sensitivity parameter *g* from the SoftMax function (Eq. 6) was the most important – small changes in the value of *g* resulted in large changes in the model fit likelihood. Thus, the mapping of associative strength to response probability is crucial in testing and development of associative models.

## Data Availability

Images, an illustrative task video, participant instructions, and data are available at the Open Science Foundation website https://osf.io/p59zu/.

## Footnotes

1 Extra-supplementary materials including images, an illustrative task video with participant instructions, code, and data are available at the Open Science Foundation website https://osf.io/p59zu/. The experiment was not pre-registered.

## Code Availability

Code for data analysis is also available at the Open Science Foundation website https://osf.io/p59zu/.
